# Harnessing the Ecological and Genomic Adaptability of the Bacterial Genus *Massilia* for Environmental and Industrial Applications

**DOI:** 10.1111/1751-7915.70156

**Published:** 2025-05-05

**Authors:** Kamyar Amirhosseini, Mehrdad Alizadeh, Hamed Azarbad

**Affiliations:** ^1^ Department of Soil Science, College of Agriculture and Natural Resources University of Tehran Tehran Iran; ^2^ Department of Plant Pathology, Faculty of Agriculture Tarbiat Modares University Tehran Iran; ^3^ Department of Biology, Evolutionary Ecology of Plants Philipps‐University Marburg Marburg Germany

**Keywords:** airborne, contaminants, *Massilia*, microbiomes, stress

## Abstract

The bacterial genus *Massilia* was first described in 1998, and since then has attracted growing interest due to its ecological plasticity and biotechnological promise. Certain species of the genus *Massilia* inhabit a variety of ecosystems, from arid deserts to polar glaciers, and exhibit unique adaptations such as resistance to cold and heat. In contaminated environments, some members of *Massilia* contribute significantly to the detoxification of heavy metals and the degradation of organic pollutants, presenting them as promising agents for bioremediation. In addition, *Massilia* species improve plant resistance and facilitate pollutant absorption in phytoremediation strategies. New research also highlights their potential as bioindicators of environmental health, given their abundance in anthropogenically influenced ecosystems and airborne microbial communities. In addition to their ecological roles, some *Massilia* species have potential in biotechnological applications by producing biopolymers and secondary metabolites. Here, we integrate findings across various habitats to present a comprehensive overview of the ecological and biotechnological importance of the genus *Massilia*. We highlight critical knowledge gaps and propose future research directions to fully harness the potential of this not fully explored bacterial genus to address environmental challenges, including contamination.

## Graphical abstract

The bacterial genus *Massilia* thrives in extreme habitats such as deserts, permafrost, and post‐fire soils. It degrades complex pollutants and collaborates with mycorrhizal fungi to degrade hydrocarbons. The ability of some members of the genus *Massilia* to produce specific secondary metabolites and biopolymers positions it as a potential candidate for industrial applications.

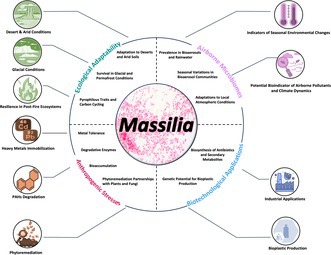

## 1 Introduction

Microbiomes play a central role in various ecosystems, where different members of microbial groups contribute to certain functions, including the degradation of pollutants, the maintenance of soil fertility, and the balance of atmospheric greenhouse gases (Delgado‐Baquerizo et al. 2018; Singh et al. 2010). Over the past decades, due to rapid advances in sequencing technologies, our knowledge of the diversity and evolution of microbiomes has greatly improved (Thomas and Segata 2019). For some of the important microbial genera, detailed mechanisms driving their diversity and adaptability have been discussed (Contreras‐Cornejo et al. 2016; Duchateau et al. 2024; Mehmood et al. 2023; Walterson and Stavrinides 2015), but less is known about others, including the bacterial genus *Massilia*.

**FIGURE 1:**
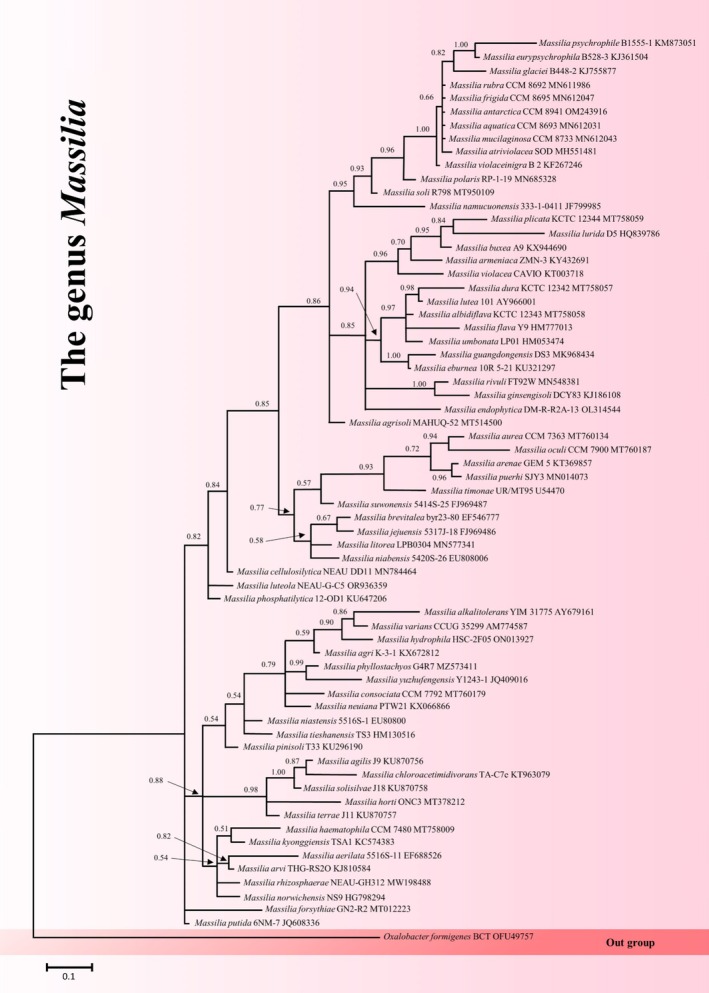
The phylogenetic trees of *Massilia* spp. including all 66 documented species. Bayesian tree inferred under the GTR + I + G model from the 16S rRNA sequences for *Massilia* spp., using *Oxalobacter formigenes* BCT OFU49757 as outgroup. The sequences were aligned using MAFFT software and subsequently refined with the Gblocks program. Clades with Bayesian posterior probabilities greater than 0.50 are indicated on the phylogenetic tree. See supplementary information for more details.

The genus *Massilia* is a member of the family *Oxalobacteraceae* within the class *Betaproteobacteria*, which was first isolated and described by La Scola et al. (1998) from the blood of a patient with variable immunodeficiency. Since then and over the last 27 years, the members of *Massilia* have been reported in various ecosystems globally, including extreme environments, such as *M. glaciei* (from the ice core in the Glacier; Gu et al. 2017) and *M. arenae* (from a desert environment; Zhang et al. 2020), polluted soils (Dahal et al. 2021; Wan et al. 2020; Wang et al. 2016), the rhizosphere of different plants (Cui et al. 2023; Yi et al. 2023), air (Li et al. 2020; Xu et al. 2023) and rain samples (Ladin et al. 2021; Péguilhan et al. 2021). *Massilia* spp. are all Gram‐negative, rod‐shaped, aerobic bacteria with a relatively high DNA G + C content, non‐spore‐forming, and motile, except for some species (Du et al. 2021; Jeon et al. 2023). Currently, there are 66 documented species within this genus (Huang et al. 2024; Lee et al. 2024), as illustrated in Figure 1. The *Massilia* genus was reported as a dominant member of soil microbial communities in fire‐affected environments (Whitman et al. 2019, 2022; Pulido‐Chavez et al. 2023; Soria et al. 2023), with studies reporting up to a 64‐fold increase in abundance in burned compared to unburned soils (Whitman et al. 2019). This bacterial genus has been recognised for its significant contributions to the biogeochemical cycling of various pollutants, including heavy metals (Hong et al. 2015; Li et al. 2014; Wan et al. 2020), organic pollutants, and hydrocarbons (Bodour et al. 2003; Li et al. 2023; Lu et al. 2019), making it a potentially crucial player in environmental remediation. Moreover, recent studies have revealed that *Massilia* is abundant in airborne microbiomes, where it accounts for up to 27% relative abundance of the microbial community in rainwater (Zhang et al. 2022) and more than 80% of the aerosol microbial composition in heavily urbanised areas (Li et al. 2020). This quantitative dominance suggests the potential of the genus *Massilia* as a bioindicator of environmental changes and stresses, which we explore in more detail in the following sections.

In addition to ecological and environmental perspectives, there is a growing interest in certain species of the genus *Massilia* due to their potential biotechnological applications. For instance, some *Massilia* isolates are capable of synthesising polyhydroxyalkanoates (PHAs), which are biodegradable microbial polyesters with potential applications in the production of bioplastics (Bassas‐Galia et al. 2012; Jeon et al. 2023). Another example is the ability to produce secondary metabolites such as violacein, a violet pigment with potent anticancer and antibacterial properties (Myeong et al. 2016). In light of the metabolic capacities of *Massilia* species and their ecological significance, a comprehensive synthesis of their ecological and biotechnological applications is needed. Such knowledge could facilitate environmental restoration practices and advance industrial biotechnology. Therefore, the main objective of this review was to integrate findings on the bacterial genus *Massilia* from diverse extreme environments (with a focus on polluted ecosystems) to present a holistic overview of its ecological roles and potential implications for environmental management. By examining the genomic and metabolic diversity of *Massilia*, this review offers insight into the potential applications of these bacteria in the context of bioremediation and biotechnology. In addition, we highlight critical knowledge gaps and suggest future research directions to harness the full potential of *Massilia* to address some of the most pressing environmental issues.

## 2 Extreme Environments

The bacterial genus *Massilia* has been detected in various extreme environments, ranging from desert and arid conditions to glacial and permafrost regions (Table 1). Flagellar motility is a key mechanism used by *Massilia* species such as *M. albidiflava* , *M. plicata* (Zhang et al. 2006), and *M. violaceinigra* (Wang et al. 2018), facilitating their movement and allowing them to navigate diverse environments effectively. Zhang et al. (2020) isolated *M. arenae* from the harsh high‐altitude soils of the Qinghai–Tibetan Plateau in China. This species was characterised by lipid profiles containing fatty acids, including C16:1 ω6c/ω7c, C16:0, and polar lipids such as diphosphatidylglycerol, phosphatidylethanolamine, and phosphatidylglycerol (Table 1). Similarly, *M. glaciei* and *M. armeniaca* , isolated from a glacier core and desert soil, respectively, displayed comparable lipid profiles, suggesting that these features may enhance membrane stability and functionality under harsh conditions (Ren et al. 2018; Gu, Liu, et al. 2017).

**TABLE 1:** Ecological roles and functional adaptations of *Massilia* species in extreme environments, including deserts, glaciers, and post‐fire ecosystems.

*Massilia*	Environmental conditions	Highlighted function	Reference
*Massilia* sp.	Soil from semi‐arid regions in Eastern Kenya	The ability to solubilise phosphate, making it a potential candidate for biofertilizer development to improve soil fertility and plant growth	Kirui et al. (2022)
*Massilia alkalitolerans*	Isolated from sandy soils in Merzouga, part of the Moroccan Sahara	Thrive in extreme desert conditions, contributing to the microbial diversity in such environments	Manni et al. (2022)
*Massilia brevitalea*	As an endophyte in *Grimmia montana* , a xerophilous moss collected from stone surfaces in the Beijing Songshan National Nature Reserve	Potential role in stress tolerance and adaptability to extreme conditions	Liu et al. (2014)
*Massilia arenae* sp. nov., strain GEM5	Sand soil samples collected from the Qinghai–Tibet Plateau in China (in a desert environment)	Major fatty acids include C16:1ω6c/ω7c and C16:0. The polar lipids include diphosphatidylglycerol, phosphatidylethanolamine, phosphatidylglycerol	Zhang et al. (2020)
*Massilia armeniaca* sp. nov., strain ZMN‐3	Soil samples collected from Inner Mongolia, China (in a desert environment)	Fatty acid profile (e.g., high amounts of C16:1 ω7c and/or C16:1 ω6c, and C16:0), and distinct polar lipids including diphosphatidylglycerol, phosphatidylglycerol, and phosphatidylethanolamine	Ren et al. (2018)
*Massilia agri* *Massilia varians*	In non‐vegetated sand dunes from Southern Algeria (characterised by arid conditions)	Adaptability to extreme arid environments, indicating its potential role in ecological processes in desert microbiomes	Selmani et al. (2023)
*Massilia glaciei* sp. nov., strain B448‐2 T	Ice core in the Muztagh Glacier, located on the Tibetan Plateau. The strain was specifically found at a depth of 23.72 m in the ice core	Unique chemotaxonomic characteristics, such as the presence of fatty acids (C16:1 ω7c and/or C16:1 ω6c, and C16:0) and polar lipids (phosphatidylethanolamine, phosphatidylglycerol, and diphosphatidylglycerol)	Gu, Liu, et al. (2017)
*Massilia frigida* strain DJPM01	From the Don Juan Pond Basin in Antarctic, within a microbial mat composed of various microorganisms	Genes for cold adaptation (e.g., cold shock proteins, RNA helicases, protein chaperones), osmotic and oxidative stress tolerance (e.g., cation/proton antiporters, catalase, peroxiredoxin), and the production of the red pigment prodigiosin, which has antimicrobial and UV‐protective properties	Shaffer et al. (2023)
*Massilia* spp.	Detected in transplanted permafrost soils from Muot da Barba Peider in eastern Switzerland	As a copiotrophic taxon, thriving in conditions with increased availability of substrates and nutrients due to warming	Perez‐Mon et al. (2022)
*Massilia* spp.	High‐temperature sediments (78°C to 98°C) samples collected from Manikaran hot springs in Himachal Pradesh, India	Genetic adaptations for thermophily and metabolic flexibility, enabling it to thrive in high‐temperature, nutrient‐limited environments	Mahato et al. (2019)
*Massilia* spp.	Soil samples collected from burned stands from semi‐arid Mediterranean forests in Almería, Spain	Dominant in the bacterial community structure post‐prescribed burns, indicating its role in early ecosystem recovery	Soria et al. (2023)
*Massilia* spp.	Soil samples collected from the Holy Fire burn area in the Cleveland National Forest, Southern California	Pyrophilous microbes, showing resilience to fire and playing a role in the initial recovery and successional dynamics of the soil bacterial community post‐fire	Pulido‐Chavez et al. (2023)
*Massilia* spp.	Soil samples from large wildfires in northern Alberta, Canada	Resilience and adaptability to fire‐affected environments, becoming a dominant genus in the bacterial community post‐fire	Whitman et al. (2019) Whitman et al. (2022)
*Massilia* spp.	The rhizosphere of holm oak forests located in the Sierra Nevada National and Natural Park, SE Spain, in areas affected by wildfires	Resilience in post‐fire conditions is linked to its xerotolerance, nitrogen mineralization, and the ability to metabolise pyrogenic carbon compounds	Fernández‐González et al. (2023)
*Massilia* spp.	The soil was from a post‐wildfire coniferous forest in Colorado	Fast growth and resource acquisition under nutrient‐scarce, high‐pH conditions, which might drive carbon losses from the soil	Nelson et al. (2024)

In the context of extremely cold environments, *M. glaciei* was isolated and characterised from an ice core in the Muztagh Glacier, located on the Tibetan Plateau, revealing its psychrophilic nature and unique chemotaxonomic characteristics that are suitable for survival in glacial conditions (Gu, Chen, et al. 2017). Similarly, Shaffer et al. (2023) extracted *M. frigida* from an Antarctic microbial mat. Their results showed genomic adaptations of this species to extreme cold and high UV radiation (e.g., the production of the antimicrobial pigment prodigiosin). Based on the soil transplantation field experiment, Perez‐Mon et al. (2022) observed that the genus *Massilia* was one of the most abundant and responsive microbial groups in alpine permafrost under experimental warming. The authors proposed that this pattern could be attributed to the role of *Massilia* as copiotrophic microorganisms that are capable of quickly adapting to higher temperatures and increased nutrient availability. This is due to the release of nutrients from previously frozen layers, allowing them to grow faster than other microbial groups. The unique capacities of members of *Massilia* to adapt and survive in various ecosystems are supported by genetic and molecular evidence. For example, analysis of clusters of O‐antigen genes, a crucial component of the bacterial outer membrane with a significant role in environmental interactions, in various species within the *Oxalobacteraceae* family showed that *Massilia* species exhibit the highest number of these genes, which could, to some extent, explain the ability of bacteria to adapt to various environmental niches (Afonnikova et al. 2022).

In several studies, *Massilia* has been reported as the dominant bacterial genus in post‐fire environments (Nelson et al. 2024; Pulido‐Chavez et al. 2023; Soria et al. 2023; Whitman et al. 2019, 2022), indicating its potential role in the resistance and resilience of soil microbial communities. Their dominance after wildfires can be potentially due to fast reproduction and their ability to take advantage of the high availability of resources (e.g., hydrocarbon compounds) after fire events. Based on the metagenomics approach, Nelson et al. (2024) studied the effects of wildfires on the soil microbiome in coniferous forests, focusing on the bacterial life history traits related to carbon cycling. Based on their findings, *Massilia* was categorised as one of those taxa with pyrophilous life history traits, which means that it is a heat‐resistant bacterial genus and thrives in severe fire‐affected environments. Its survival was attributed to its fast growth rate and high resource acquisition traits, allowing it to dominate with limited nutrients under nutrient‐scarce, high pH conditions. Additionally, *Massilia* exhibits resilience by using necromass (dead organic matter) as a resource, an important adaptation strategy in post‐fire soils where organic matter from burnt vegetation is abundant (Nelson et al. 2024). While these findings position *Massilia* as a key player in the early stages of microbial succession in fire‐affected soils, the broader ecological and biogeochemical consequences remain underexplored. For instance, the accelerated growth and metabolic activity of *Massilia* may contribute to increased soil carbon losses through microbial respiration, potentially influencing carbon dynamics in fire‐impacted ecosystems. However, it is not clear how members of *Massilia* compare with other pyrophilous taxa in terms of their contribution to soil carbon turnover. Addressing these questions could help to understand better the role of soil microbiomes in post‐fire carbon cycling and improve predictions of carbon fluxes in response to the increasing frequency and severity of wildfires.

## 3 Bioremediation and Pollution Response

Microbiomes play an important role in the resistance and resilience response of different ecosystems in the face of stressors such as those associated with pollutants (Azarbad, van Gestel, et al. 2016; Azarbad, van Straalen, et al. 2016; Azarbad et al. 2015). Among microbes, *Massilia* has emerged as a keystone genus capable of adapting to a wide range of pollutants and contributing to their degradation (Tables 2 and 3). In this part, we review the research on the role of *Massilia* in pollutant degradation and its potential in bioremediation strategies.

**TABLE 2:** Contributions of *Massilia* species to the biodegradation of heavy metals and organic pollutants in industrially and environmentally degraded ecosystems.

Pollutant	*Massilia*	Environmental/experimental conditions	Highlighted functions	References
Cd, Cr, Cu, Zn, and Fe	*Massilia* spp.	Iron mining areas with varying levels of heavy metal pollution	Dominant genus in heavily polluted soils, demonstrating its resilience to high levels of metals	Hong et al. (2015)
As	*Massilia* spp.	Groundwater samples from Mongolia	Dominant genus in arsenic‐rich groundwater samples, suggesting its role in arsenic, iron, and nitrogen cycling	Li et al. (2014)
Cd	Massilia spp.	Paddy fields with Cd concentrations ranging from 0.24 to 9.55 mg/kg	Cd immobilisation through ureolysis, forming CdCO_3_ precipitates	Li et al. (2024)
Hg	*Massilia* spp.	A mercury smelting enterprise. The soils were contaminated with varying Hg levels (0.5, 5, and 500 mg/kg)	Substantial increase in abundance and contributing to mercury detoxification	Wan et al. (2020)
Ni, Mn	*Massilia* sp. strain Mn16‐1_5	Isolated from soil with high metal content	A high potential for bioremediation through manganese and chromium detoxification	Chen et al. (2019)
Pb	*Massilia plicata* (gp‐3), *Massilia lutea* (gp‐6)	Isolated from agricultural soils exposed to heavy metal contamination, particularly lead	Phosphorus solubilisation and lead immobilisation in contaminated soils	Wan et al. (2020)
PAHs	Massilia spp.	A coal‐power plant's surrounding and amended with corn straw and citric acid	Enhancing PAH degradation efficiency in amended soils	Bao et al. (2021)
Phenanthrene	*Massilia* sp. WF1	This study explores a novel bioremediation approach by utilising the fungal mycelia of *Phanerochaete chrysosporium* to facilitate the migration of *Massilia*	Massilia sp. WF1 adhered to fungal mycelia of *Phanerochaete chrysosporium*, facilitating its migration and significantly enhancing phenanthrene degradation	Gu, Chen, et al. (2017)
Methomyl and triazophos	*Massilia* spp.	Contaminated agricultural soil with methomyl (0.11 mg/kg), triazophos (0.02 mg/kg), and heavy metals	Dominant genus contributing to the degradation of pesticides such as methomyl and triazophos	Kroeksakul et al. (2023)
Phenanthrene	*Massilia* spp.	Petroleum‐contaminated soil with 36.32 mg/kg phenanthrene	Cooperative metabolism with fungi to degrade phenanthrene	Li et al. (2021)
PAHs	*Massilia* sp. WG5	Isolated from phenanthrene‐contaminated soil	Contains genes related to xenobiotic biodegradation and PAH metabolism	Lou et al. (2016)
PAHs	*Massilia* sp. WF1	Isolated from PAH‐contaminated soil under varying pH, temperature, and phenanthrene concentrations	Rapid degradation of phenanthrene, converting it into non‐toxic intermediates	Wang et al. (2016)
BTEX (benzene, toluene, ethylbenzene, and xylene)	*Massilia aromaticivorans* sp. nov. (ML15P13T)	Isolated from Arctic soil in Svalbard Islands, Norway	Degradation of BTEX compounds via the β‐ketoadipate pathway, producing enzymes that break down aromatic hydrocarbons	Son et al. (2021)
Oil	*Massilia antibiotica* sp. nov. (strain TW‐1 T)	Isolated from oil‐contaminated soil	Antimicrobial activity against *Escherichia coli* and *Pseudomonas aeruginosa*	Dahal et al. (2021)
Diesel hydrocarbons	*Massilia* spp.	Sub‐Arctic diesel‐contaminated soils from Northern Quebec, Canada	Key hydrocarbon‐degrading genera, contributing to bioremediation in cold environments	Kundu et al. (2023)
Phenanthrene	*Massilia* sp. WF1	This study investigates the role of *Massilia* and *Phanerochaete chrysosporium* in phenanthrene biodegradation	High phenanthrene degradation capacity	Gu et al. (2021)
Oil	*Massilia* spp.	Heavy oil‐contaminated soil with 5 g/kg heavy oil	Enhanced bioremediation	Lv et al. (2023)
Oil	*Massilia* spp.	Soil from oil well areas, spiked with 15 g/kg, 30 g/kg, and 45 g/kg oil concentrations	Enriched in oil‐contaminated soils areas	Shi et al. (2022)
PAHs	*Massilia* spp.	Bioretention facility soils from Shaanxi Province, China, sampled during rainy and non‐rainy seasons; soil was exposed to PAHs	Strong positive correlations with naphthalene (NAP) and fluoranthene (FLT)	Li et al. (2023)
Phenanthrene, Fluoranthene, Pyrene	*Massilia* spp.	Urban soil from Nanchong, China, with added phenanthrene, fluoranthene, and pyrene (2 μg/g each), incubated with/without rhamnolipid (20 μg/g)	Dominant hydrocarbon‐degrading bacterium	Lu et al. (2019)
Decabromodiphenyl ether (BDE209) and Pb	*Massilia* spp.	Soil from an e‐waste recycling site in Taizhou, Zhejiang, China, spiked with 10 mg/kg BDE209 and 560 mg/kg Pb	Enriched in contaminated soils	Zhang et al. (2016)
PAHs, PCBs, and heavy metals	*Massilia* spp.	Surface soils from Guiyu, an e‐waste processing center, spiked with phenanthrene and pyrene for lab incubation	Enriched in PAH‐contaminated soils at an e‐waste processing center	Zhang et al. (2010)
Phenanthrene	*Massilia timonae*	Soil exposed to phenanthrene (1.2 mg/L) for 6 months	Key phenanthrene degraders	Bodour et al. (2003)
PAHs	*Massilia* spp.	Soil from agricultural areas near a steel plant with long‐term PAH exposure: phenanthrene (47.36 mg/kg), pyrene (50.13 mg/kg), and benzo(a)pyrene (9.66 mg/kg)	PAH degradation	Li et al. (2019)

*Note:* **Methomyl**: A carbamate pesticide commonly used in agriculture to control pests. **Triazophos**: An organophosphate insecticide used to manage pests in crops. **PAHs (Polycyclic Aromatic Hydrocarbons)**: A group of organic compounds produced from incomplete combustion of organic materials like coal, oil, and wood. **Decabromodiphenyl Ether (BDE209)**: A type of polybrominated diphenyl ether (PBDE) used as a flame retardant in electronics, textiles, and plastics. **BTEX (Benzene, Toluene, Ethylbenzene, and Xylene)**: Volatile organic compounds in petroleum products. **PCBs (Polychlorinated Biphenyls)**: Industrial chemicals used in electrical equipment and other applications. **Phenanthrene**: A type of PAH commonly found in coal tar and crude oil.

**TABLE 3:** Roles of *Massilia* in plant‐microbe interactions: Enhancing phytoremediation, plant growth, and soil health in polluted and contaminated soils.

Pollutant type	Massilia	Environmental/experimental conditions	Plant association	Highlighted functions	References
As, Cd	*Massilia* spp.	A controlled pot experiment with artificially spiked soils	Rhizosphere of the Chinese brake fern ( *Pteris vittata* )	Increase in the relative abundance in the rhizosphere	Cui et al. (2023)
Cd	*Massilia timonae*	Cd‐polluted soil due to irrigation with sewage water for over 20 years	Rhizosphere of *Solanum nigrum* L.	Cd mobilisation, contributing to soil acidification and increasing Cd bioavailability for plant uptake	Xu et al. (2020)
Cu, Cd, As	*Massilia* spp.	The study explored bacterial communities in *Bothriochloa ischaemum* litter over years of phytoremediation in a copper tailings dam	Litter of *Bothriochloa ischaemum*	Litter decomposition, enzyme activity (e.g., cellulase and catalase), and the stabilisation of metals in the soil	Jia et al. (2020)
Cd, Pb, Zn, PAHs, and PCDDs	*Massilia niastensis*	The role of rhizobacterial inoculants in remediating complex contaminant mixtures in urban bottom sediments	Rhizosphere of *Festuca rubra*	Resistance to Cd and Zn, promoting plant growth via IAA production and increasing plant biomass	Siebielec et al. (2019)
Cd and PAHs	*Massilia* sp.	Soils were contaminated with cadmium (Cd) and PAHs due to long‐term irrigation with contaminated water	Rhizosphere of *Oryza sativa* (rice)	Enhances the degradation of phenanthrene and facilitates the reduction of bioavailable heavy metals	Yi et al. (2023)
Cd	*Massilia* sp. III‐116‐18	The bacteria were isolated from rhizosphere soils and ectomycorrhizae of *Salix viminalis* growing on Cd‐contaminated, anthropogenically degraded sites	Rhizosphere of *Salix viminalis*	Bioaccumulation and intracellular distribution of Cd	Hrynkiewicz et al. (2015)
As, Cd	*Massilia* spp.	Soils were contaminated with As (410 mg/kg) and Cd (3 mg/kg)	Rhizosphere of *Sedum alfredii*	Enhancing cadmium phytoextraction, contributing to microbial diversity under heavy metal stress	Wang et al. (2022)
Fe, Mn, Ti	*Massilia* spp.	The role of microbial populations in the root systems of native plants during the natural attenuation of V–Ti magnetite mine tailings	Rhizosphere of *Bothriochloa ischaemum*	Enriched in the rhizosphere	Kang et al. (2020)
PAHs and alkanes	*Massilia* spp.	This study explored the bacterial diversity associated with AMF propagules within roots of *Solidago rugosa* growing in petroleum hydrocarbon‐polluted soils	Rhizosphere of *Solidago rugosa*	Dominant bacterial genera associated with AMF, playing a potential role in hydrocarbon degradation	Iffis et al. (2014)
DEHP, Cd, and Zn	*Massilia* sp.	This study explores the potential of phytoremediation using rice and PGPR consortium for the remediation of DEHP and heavy metal co‐contaminated soils	Rhizosphere of *Oryza sativa* (rice)	Immobilising Cd and Zn, significantly enhancing pollutant removal from the soil while promoting plant growth	Liu et al. (2022)
Pyrene	*Massilia* spp.	Rhizosphere sediments from *Vallisneria natans* , collected from sediment spiked with pyrene	Rhizosphere of *Vallisneria natans*	Degrading pyrene in aquatic rhizosphere sediment	Ge et al. (2023)
Phenanthrene	*Massilia* sp. Pn2	Soil contaminated with phenanthrene (2.23, 30.24, 134.64 mg/kg)	Roots of Wheat ( *Triticum aestivum* L. cv. Yangmai‐16)	Reduced phenanthrene levels in both wheat roots and shoots, while also promoting root growth and altering the endophytic bacterial community structure.	Liu et al. (2017)
Microplastics and phenanthrene	*Massilia* spp.	Soil contaminated with phenanthrene (PHE) at a concentration of 150 mg/kg. Two types of polyethylene microplastics (D550: 550 μm and D250: 250 μm) were added at 2% (w/w)	Rhizosphere of maize ( *Zea mays* L.)	Microplastic contamination reduced the abundance of *Massilia*	Chen et al. (2023)

*Note:* **PCDDs (Polychlorinated Dibenzo‐p‐Dioxins)**: PCDDs are a group of chemically related compounds often called “dioxins”. They are byproducts of industrial processes such as waste incineration, chemical manufacturing, and pesticide production. **DEHP (Di(2‐ethylhexyl) phthalate):** DEHP is a commonly used phthalate plasticizer, primarily added to plastics like PVC to increase flexibility.

### 3.1 Heavy Metal Contamination

*Massilia* has been reported to be involved in bioaccumulation and immobilisation of several toxic metals in contaminated environments. Previous studies have demonstrated the ability of *Massilia* to reduce the bioavailability of cadmium (Cd), lead (Pb), mercury (Hg), and nickel (Ni) through several mechanisms (Tables 2 and 3). For example, this bacterial genus has been shown to immobilise Cd by inducing its precipitation as stable carbonate forms, significantly reducing its mobility in soil matrices (Li et al. 2024). Furthermore, *M. plicata* and *M. lutea* have been reported to immobilise Pb in heavily polluted agricultural soil (Wan et al. 2020). *Massilia* sp. (strain III‐116‐18), isolated from rhizosphere soils of willows grown on Cd‐contaminated sites, demonstrated high Cd bioaccumulation efficiency through sequestering Cd^2+^ intracellularly and in the cell wall (Hrynkiewicz et al. 2015). Hong et al. (2015) studied the effect of heavy metal pollution on soil bacterial communities in iron mining areas (containing elevated levels of Cd, Cr, Cu, and Zn) where *Massilia* was reported as a dominant genus. Similarly, Zhang et al. (2016) demonstrated that *Massilia* was enriched in Pb‐contaminated soils (560 mg/kg Pb). These findings suggest that the metal immobilisation capacity, as well as metal tolerance, makes *Massilia* a suitable candidate for bioremediation technologies aimed at restoring contaminated ecosystems.

### 3.2 Polycyclic Aromatic Hydrocarbons (PAHs)

PAHs, which are persistent organic pollutants, present significant environmental risks due to their toxicity and resistance to degradation (Gu et al. 2021; Li et al. 2021, 2019). The low solubility of such compounds (e.g., phenanthrene and pyrene) is one of the major obstacles in bioremediation efforts, further hindering microbial degradation. Despite these challenges, several studies have reported *Massilia* as one of the major contributors to the degradation of PAHs (Tables 2 and 3). For instance, a recent study conducted by Li et al. (2023) reported *Massilia* as a PAH‐tolerant bacterium enriched in response to naphthalene and fluoranthene. Similarly, Wang et al. (2016) showed that *Massilia* sp. (strain WF1 isolated from PAH‐contaminated soil) exhibited rapid degradation of phenanthrene, converting it into non‐toxic intermediates. The authors showed that the biodegradation process is initiated by dioxygenase‐mediated cleavage of phenanthrene's aromatic rings, producing metabolites such as 1‐hydroxy‐2‐naphthoic and phthalic acids. Another line of evidence on the molecular degradation mechanisms employed by *Massilia* sp. (strain WF1) is the study conducted by Gu et al. (2021). The authors revealed that degradation mechanisms likely involve specific genes, such as PAH‐RHDα GP and nidA, which are associated with the production of enzymes, like oxygenases, that are capable of initiating the breakdown of PAH compounds. The ability of *Massilia* to degrade PAHs positions it as an important microbial tool in the bioremediation of PAH‐contaminated environments.

### 3.3 Plant–Microbe Interactions Under Heavy Metal and Organic Contaminants

In the context of plant‐microbe interactions in the presence of heavy metals and organic pollutants, *Massilia* species contribute to enhancing plant responses and facilitating the degradation of pollutants (Table 3). For example, members of *Massilia* isolated from the rhizosphere of plants, such as *Pteris vittata* and *Oryza sativa* , have been found to be dominant in the rhizosphere, potentially contributing to reducing the toxicity and bioavailability of metals such as Cd and As (Cui et al. 2023; Yi et al. 2023). Another example is *M. niastensis* , which plays a dual role in resistance to heavy metals and producing plant growth‐promoting hormones such as indole‐3‐acetic acid (IAA), which supports biomass accumulation in *Festuca rubra* under complex contamination involving heavy metals and organic pollutants (Siebielec et al. 2019). By colonising the rhizosphere and interacting with plant roots, *M. timonae* enhances Cd mobilisation within the rhizospheric environment, facilitating its uptake and subsequent remediation by plants (Xu et al. 2020). Liu et al. (2017) demonstrated that *Massilia* sp. (strain *Pn2*), an endophytic bacterium isolated from wheat, significantly reduced phenanthrene levels in wheat roots and shoots. The authors also highlighted the ability of *Massilia* to alter the structure of the endophytic bacterial community in response to phenanthrene stress, linked with the improvement of plant resilience. Such interactions not only promote pollutant removal but also stimulate plant growth by altering the structure of the microbial community and enhancing enzyme activities such as cellulase and catalase, contributing to soil stabilisation and nutrient cycling (Jia et al. 2020).

### 3.4 Plant‐Microbe Interactions Under Microplastic Contamination

Due to their persistence and small particle size, the accumulation of microplastics in soil ecosystems, particularly in agricultural soils, presents a significant threat to soil biota and overall soil health (Sun et al. 2022; Xiang et al. 2024). Xiang et al. (2024) explored the effects of polystyrene microplastics on the rhizosphere microbial community and agronomic traits of barley. Their results revealed significant shifts in rhizosphere communities, particularly an increase in the relative abundance of the bacterial genus *Massilia*, *Ralstonia*, and *Achromobacter*. The opposite observation was made by Chen et al. (2023) who studied the combined effects of two types of polyethylene microplastics and phenanthrene on the rhizosphere microbial communities of maize. Their results showed that microplastics decreased the abundance of *Massilia*, a key PAH‐degrading bacterium, which reduced the removal of phenanthrene from the soil. Similarly, using wheat as a model plant, the combination of oxytetracycline (as an antibiotic) and polyethylene microplastic exposure led to a significant reduction in the relative abundance of *Massilia* in the rhizosphere, contributing to reduced wheat growth (Guo et al. 2022). These contrasting findings suggest that the response of *Massilia* to microplastics is context‐dependent, depending on the microplastic type, co‐contaminants, and association with specific plants. Understanding the dynamics of *Massilia* and its interactions with plants and other members of soil microbes is important for developing strategies to mitigate the adverse effects of microplastics on soil health and agricultural productivity. Future research should focus on elucidating the mechanisms underlying *Massilia*'s responses to microplastic contamination, including its potential role in pollutant degradation in the disturbed soil and rhizosphere environments.

### 3.5 Microbe‐Microbe Interactions Under Heavy Metal and Organic Contaminants

Microbe‐microbe interactions, particularly between *Massilia* species and fungi, have been shown to be important in the degradation of contaminants in polluted environments. Iffis et al. (2014) studied the bacterial diversity associated with arbuscular mycorrhizal fungi (AMF) within the roots of *Solidago rugosa* that grows in petroleum hydrocarbon‐polluted soils. *Massilia* was identified as one of the dominant bacterial genera associated with AMF, playing a potential role in hydrocarbon degradation. However, the authors noted that the impact of *Massilia* on AMF remains uncertain. Another line of evidence highlighting *Massilia*'s ability to degrade pollutants through association with fungi is a study conducted by Gu, Chen, et al. (2017). They demonstrated the interaction between *Massilia* sp. (strain *WF1*) and the hydrophobic fungus *Phanerochaete chrysosporium*, where the bacterial strain passively adhered to fungal hyphae and migrated across the soil, facilitated by flagella and a type III secretion system. This fungal‐assisted migration significantly enhanced phenanthrene biodegradation by improving the spatial distribution of *Massilia* in the soil and increasing contact with hydrophobic organic compounds. A similar observation was made by Li et al. (2021), who reported the synergistic interaction between *Massilia* and fungi, which contributed to improved bioremediation of soils contaminated with phenanthrene. Such interactions between *Massilia* and fungal hyphae open several questions: What are the molecular mechanisms behind such interactions? Who controls and initiates these interactions, fungi or bacteria? What is the advantage of such an association for fungi? In general, limited information is available on bacterial species that inhabit fungal hyphae (Basiru et al. 2023; Sangwan and Prasanna 2022; Scheublin et al. 2010).

## 4 Airborne Microbiomes

The airborne microbiome has recently received significant attention due to its crucial role in atmospheric processes, such as cloud formation, precipitation, biogeochemical cycles (DeLeon‐Rodriguez et al. 2013; Šantl‐Temkiv et al. 2022), and public health (Li, Feng, et al. 2024; Moelling and Broecker 2020; Xu and Yao 2020). Previous studies have reported *Massilia* as a dominant group of atmospheric microorganisms (Table 4).

**TABLE 4:** Summary of studies on *Massilia* as the dominant species of airborne microbiomes.

Environmental/experimental conditions	*Massilia*	Remarks	Reference
Air samples from a constructed wetland ecosystem (Qingdao, China)	*Massilia alkalitolerans* , *Massilia albidiflava* , *Massilia aurea* , *Massilia brevitalea* , *Massilia timonae*	Community structure and diversity showed seasonal variation (summer > other seasons) and were significantly influenced by temperature and humidity	Xu et al. (2023)
Microbiota carried by fluffy catkins from willow and poplar trees (Beijing, China)	*Massilia* spp.	The most abundant genus carried by the fluffy catkins in two out of the three locations under study	Xu and Yao (2020)
Urban dust‐associated bacterial community (dust samples collected from five functional district in Beijing, Tianjin, and Shijiazhuang, China)	*Massilia* spp.	The abundance was significantly higher in the summer than in the winter	Zhou et al. (2018)
Aerosols from different land types (forest, lake, garden, wetland, urban street, bare soil, cropland, livestock farm, sewage treatment plant, and smelter factory in Beijing, China)	*Massilia* spp.	Dominant genus in areas with high levels of anthropogenic activities such as cropland, sewage plant, smeltery, and urban streets, indicating potential roles as a bioindicator	Li et al. (2020)
Aerosols from dairy farms (collected from two dairies located 190 km apart, in Sonoma and Modesto, California, USA)	*Massilia albidiflava* *Massilia aerolata Massilia* sp. (acc. no. HM437163) *Massilia* sp. (acc. no. EU626407) *Massilia* sp. (acc. no. GU933592)	Abundant in the aerosols but undetected in fresh and dry manures	Ravva et al. (2011)
Rainwater samples (Hulunbuir Grassland Ecosystem Research Station of the Chinese Academy of Agricultural Sciences at Xiertala Farm, Inner Mongolia, China)	*Massilia* spp.	Potential role as bioindicators of changes in the local climatic conditions	Zhang et al. (2022)
Cloud water, rainwater, and snow samples (collected from meteorological stations in puy de Dome Mountain, France)	*Massilia* spp.	Among the most abundant genera in cloud water, rainwater, and snow samples, with the genus more represented in rain than in other atmospheric samples	Péguilhan et al. (2021)
Throughfall and rainwater samples (collected from a 16‐ha forest in Newark, Delaware, USA)	*Massilia* sp.	*Massilia* sp. was found in both throughfall and rainwater samples, with a notable prevalence in throughfall samples	Ladin et al. (2021)

In a constructed wetland ecosystem, *Massilia* spp. were identified in the air samples, with seasonal fluctuations where greater abundance and richness were observed in summer than in the other seasons (Xu et al. 2023). The study revealed that air conditions, such as temperature, humidity, wind speed, and particulate matter (PM2.5 and PM10), may influence the seasonal variation and community structure of airborne *Massilia*. Importantly, opportunistic pathogenic species such as *M. timonae* and *M. oculi* were detected in air samples, raising public health concerns about the airborne spread of pathogenic strains (Xu et al. 2023). Studies in urban environments further support the seasonality of *Massilia* in atmospheric systems. For instance, Zhou et al. (2018) reported that the abundance of *Massilia* at the genus level exhibited seasonal variation, with its abundance in dust samples from Beijing and Shijiazhuang in China being significantly higher in summer than in winter, likely driven by increased wind speeds and dust mobilisation. While the seasonal trends are documented, the mechanisms underlying the interactions of *Massilia* with these environmental factors are still unclear. For instance, does its seasonal abundance correlate with specific functional adaptations, or is it merely a reflection of its dispersal efficiency?

Air pollution has been shown to play a role in shaping *Massilia*'s atmospheric dynamics. For example, *Massilia* was reported among the top 20 genera in microbial aerosols emitted during air pollution characterised by high levels of fine particulate matter (PM) in Beijing, China (Zhang et al. 2019). At high PM levels (up to 208 μg/m^3^), *Massilia* was one of the most abundant taxa in microbial aerosol communities under polluted conditions. Li et al. (2020) investigated bioaerosol emissions from 13 different land types with varying degrees of human impact. They found that *Massilia* is the dominant bacterial genus in bioaerosols emitted from areas heavily affected by human activities, such as a sewage treatment plant, cropland, a smelter factory, and an urban area in Beijing, China. For instance, in highly urbanised areas like streets in Beijing, the relative abundance of *Massilia* accounted for more than 80% of the total bacterial community. These findings suggest a strong link between *Massilia* and human‐impacted environments, but the implications for air quality and public health are something that need more attention. Important questions that now need to be answered are: how does *Massilia* contribute to or mitigate the effects of air pollution, and are its adaptations unique compared to other genera prevalent under similar conditions?

*Massilia* has also been detected in rainwater, representing a major component of the bacterial community (Péguilhan et al. 2021; Zhang et al. 2022; Ladin et al. 2021). An example of this is the study conducted by Zhang et al. (2022), who reported an average relative abundance of 27% in rainwater samples collected from 2018 to 2020 (before and after the COVID‐19 epidemic). The authors hypothesised that *Massilia* may exhibit high‐efficiency ice nucleation activity (INA), a process where microbial particles serve as nuclei for ice crystal formation in clouds, which may impact the local precipitation. However, this hypothesis remains unconfirmed, as no direct experimental evidence was provided on the role of *Massilia* in these processes. Woo and Yamamoto (2020) offered complementary insights, suggesting that bacterial aggregation and attachment to larger particles enhance sedimentation velocities and nucleation efficiencies, with *Massilia* potentially contributing to cloud formation due to its large aerodynamic diameters (8.79 μm) and high sedimentation velocities (5.7 cm/s). While the genus is detected in air, dust, and rainwater samples, the mechanisms driving its interactions with atmospheric particles, pollutants, and climate‐relevant processes are yet to be fully elucidated. Future research should prioritise isolating *Massilia* strains from rainwater to experimentally test their ice nucleation activity and assess their contributions to cloud formation. Incorporating metagenomic and metatranscriptomic approaches could provide deeper insights into the functional capabilities of airborne *Massilia* and its interactions with environmental factors, such as particulate matter. Furthermore, exploring the sources and dispersal pathways of *Massilia* could reveal potential links between terrestrial, aquatic, and atmospheric microbial processes.

## 5 Biotechnological Applications

*Massilia* species have been shown to possess biosynthesis and degradation abilities, making them promising candidates for biotechnological applications. One interesting example is *Massilia* sp. (strain NR 4–1), isolated from the rhizosphere of *Torreya nucifera*. Its complete genome sequencing revealed a full violacein biosynthesis pathway, suggesting its ability to produce violacein, a compound with significant antibiotic and anticancer properties (Myeong et al. 2016). Furthermore, *M. niastensis* has been found to harbour genes responsible for the synthesis of antibacterial compounds, such as the N‐acyl amino acid synthase gene, which confers antibacterial properties against various pathogens such as *Listeria monocytogenes* and *Staphylococcus epidermidis* (Lee et al. 2019).

Members of the *Massilia* species have been reported to produce and accumulate aliphatic polyesters (i.e., polyhydroxyalkanoates; PHAs) as intracellular granules using a variety of easily accessible substrates. These polymers offer cost‐effective and biodegradable alternatives to petrochemical‐derived thermoplastics that threaten the environment and human health. Jeon et al. (2023) revealed the presence of PHA‐associated genes in *M. endophyticus* isolated from the root tissues of the plant ( *Cannabis sativa* ). In this bacterium, biosynthesis of polyhydroxybutyrate (PHB), which is a member of the PHA, begins with the condensation of two acetyl‐CoA molecules into acetoacetyl‐CoA by a β‐ketothiolase (PhaA). The acetoacetyl‐CoA is then reduced to (R)‐3‐hydroxybutanoyl‐CoA by an NADPH‐dependent acetoacetyl‐CoA reductase (PhaB), and finally a PHA synthase (PhaC) polymerises the 3‐hydroxybutanoyl‐CoA monomers into PHB. Bassas‐Galia et al. (2012) further revealed that *M. plicata* (isolated from the phyllosphere of plants) is an efficient PHB synthesiser. The strain accumulated large amounts of PHB (~50% relative to the dry weight) using the cost‐effective glycerol substrate as a carbon source. The strain yielded more PHB than most commonly recognised PHA producers while exhibiting non‐pathogenicity (Bassas‐Galia et al. 2012). Similarly, Han et al. (2014) identified *Massilia* sp. (strain UMI‐21) isolated from the green algae *Ulva* as capable of producing PHA using starch, maltose, and maltotriose as carbon sources.

However, it is important to note that PHB production is not a unique feature that belongs exclusively to *Massilia*, and many other bacterial genera are also capable of PHB production (Reddy et al. 2003). In addition, *Massilia* strains may not necessarily be the ideal hosts in terms of PHB production efficiency. Jiang et al. (2023) demonstrated this by constructing a recombinant *Escherichia coli* DH5α strain with PHB‐synthesising genes from *Massilia* sp. UMI‐21. The engineered *E. coli* strain exhibited substantially higher yields, producing up to 213.30% more PHB than the native *Massilia* strain when supplemented with glucose as the carbon source. These findings may indicate that the importance of *Massilia* in bioplastic production resides in its genetic makeup and metabolic adaptability as a source of valuable biosynthetic pathways, which may be transferred to more efficient production strains. Moving forward, future research should explore the metabolic versatility of *Massilia* through genetic engineering and its broader biotechnological applications beyond bioplastic production, which could lead to the development of new compounds. Moreover, a deeper understanding of the ecological roles of *Massilia* in diverse environments—ranging from rhizospheres to aerosols—could reveal synergistic interactions or niche‐specific adaptations that may support co‐culture‐based bioproduction systems. To achieve these goals, interdisciplinary approaches that combine microbial ecology, genomics, and bioprocess engineering are important to advance the application of *Massilia* to address pressing challenges, from antibiotic resistance to environmental sustainability.

## 6 Concluding Remarks and Future Directions

**FIGURE 2:**
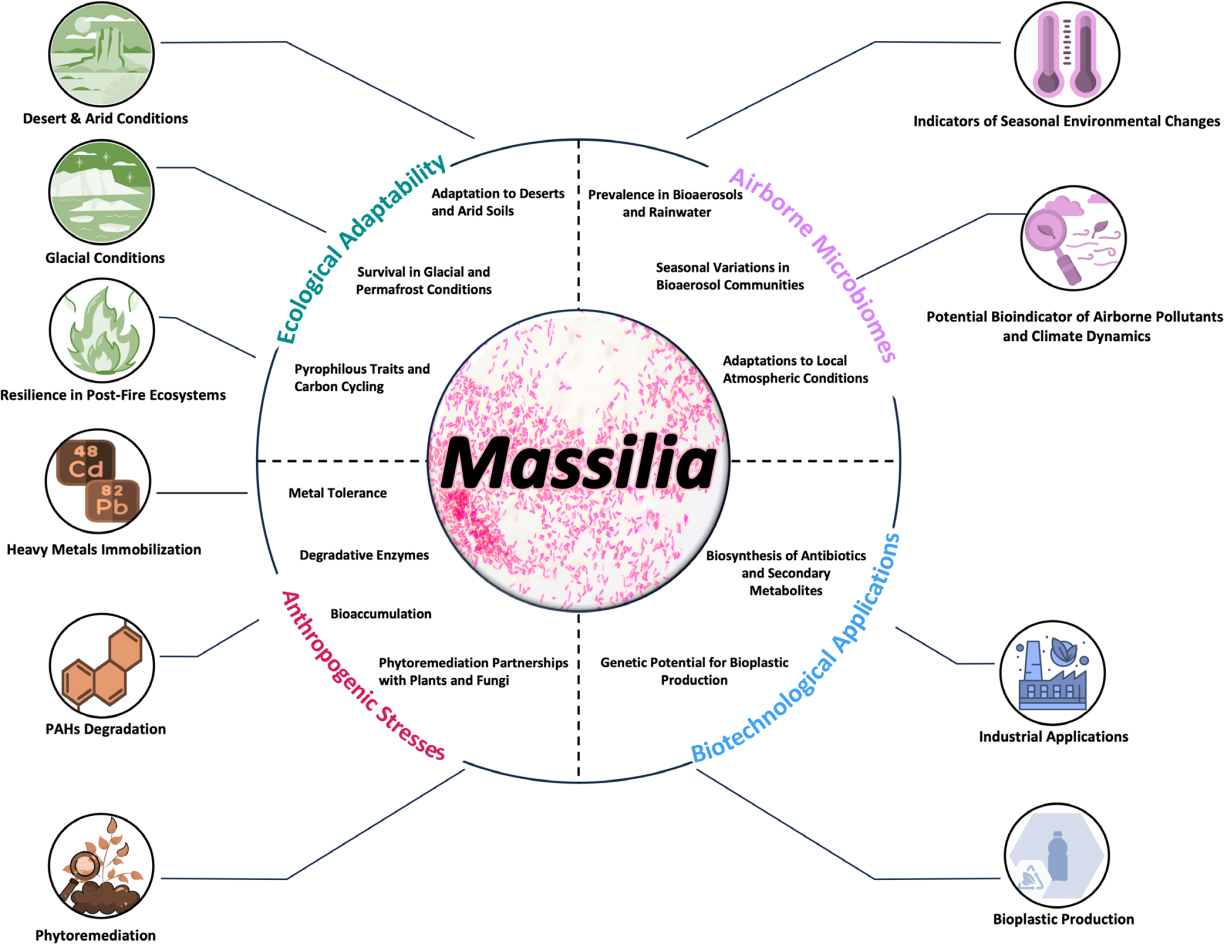
Ecological roles and adaptability of *Massilia* and the bacteria's environmental and industrial applications. This figure has been created using resources designed by Flaticon (https://www.flaticon.com).

Based on the information compiled in this review, *Massilia* exhibits a functionally diverse group of bacteria with remarkable abilities to survive in extreme environments and play ecological roles in various ecosystems (Figure 2). In extreme environments, *Massilia* species have demonstrated their ability to thrive in diverse and harsh conditions, ranging from deserts and permafrost regions to glacial environments and post‐fire ecosystems. This resilience is attributed to their unique genomic adaptations, such as genes for cold adaptation, tolerance to UV radiation, and rapid growth in response to environmental changes. Their role in post‐fire ecosystems, particularly in soil recovery and carbon cycling, highlights their ecological importance in these environments. Their ability to adapt quickly to high pH, nutrient‐rich conditions after wildfires suggests that the genus *Massilia* may contribute to accelerated carbon losses, further underlining its impact on ecosystem processes. Their metabolic flexibility, including the use of dioxygenases to degrade complex organic pollutants, makes them valuable in environmental restoration strategies. The association of members of *Massilia* with AMF further enhances its role in facilitating hydrocarbon degradation, emphasising the importance of microbial partnerships in complex environmental processes. Further studies could investigate the specific molecular mechanisms and signalling pathways involved in the interaction between *Massilia* and fungi (e.g., the adhesion and migration process). Furthermore, exploring the potential impact of environmental factors, such as different concentrations of pollutants and soil characteristics, on the efficiency of bacterial migration using fungi as carriers could provide practical insights to optimise bioremediation strategies.

The detection of the genus *Massilia* in aerosols, rainwater, and dust samples from various regions suggests its potential as a bioindicator for air quality. Further research is essential to elucidate how *Massilia* interacts with other microbial aerosols and atmospheric particles and if these interactions affect climate‐relevant processes like precipitation and air quality. We suggest refining the use of the bacterial genus *Massilia* as indicators of ecosystem health and anthropogenic disturbances, particularly in urban and industrial regions. The ability of the genus to produce some specific secondary metabolites and biodegradable biopolymers positions it as a potential candidate for industrial applications (Figure 2). Genetic engineering approaches may further enhance the yields of these bioproducts, providing sustainable alternatives to conventional industrial processes. By exploring these directions, further research can unlock the full potential of this not fully explored bacterial genus to contribute to environmental microbiology and biotechnology.

## Author Contributions

**Kamyar Amirhosseini:** investigation, writing – original draft, writing – review and editing, visualization, validation. **Mehrdad Alizadeh:** investigation, writing – original draft, writing – review and editing, visualization, validation, methodology. **Hamed Azarbad:** conceptualization, investigation, writing – original draft, writing – review and editing, visualization, validation, methodology, supervision, resources.

## Conflicts of Interest

The authors declare no conflicts of interest.

## Supporting information


Data S1.

## Data Availability

Data sharing is not applicable to this article as no datasets were generated or analysed during the current study.
