# Supplementary material for: Harnessing the Ecological and Genomic Adaptability of the Bacterial Genus Massilia for Environmental and Industrial Applications
Source: Microb Biotechnol. 2025 May 5;18(5):e70156. doi: 10.1111/1751-7915.70156 (PMC12053321; doi:10.1111/1751-7915.70156)
Supplement: Supplementary file 1 — Data S1. [file MBT2-18-e70156-s001.docx]

**The phylogenetic trees of *Massilia* spp.**

This study aimed to analyze 16S rRNA sequences obtained from LPSN (https://lpsn.dsmz.de/genus/massilia) and recent publications (Oren & Göker, 2024; Huang et al., 2024; Lee et al., 2024). The sequences were aligned using the Q-INS-i algorithm of MAFFT v.7.205, ensuring high alignment quality (Katoh & Standley, 2013). To refine these alignments, we utilized the Gblocks program (version 0.91b) with less stringent parameters to remove poorly aligned regions and gaps within blocks, enhancing the dataset's robustness for phylogenetic analysis. We selected the best-fitting models for the dataset using PAUP*/MrModeltest.2 (Nylander, 2004). Subsequently, we conducted Bayesian analyses with MrBayes 3.1.2, employing a starting random tree and the GTR+I+G model for the 16S rRNA sequences, running for four million generations to ensure convergence and accuracy. For visualization, we employed Dendroscope V.3.2.8 to create phylogenetic trees, while PowerPoint was utilized to enhance the resolution of the final output (Huson & Scornavacca, 2012). This workflow facilitated a comprehensive understanding of the evolutionary relationships within our dataset, contributing valuable insights into bacterial taxonomy.

**References**

Huang X, Qi S, Song W, Yu X, Zhang H, Xiang W, Zhao J, Wang X. Massilia luteola sp. nov., a novel indole-producing and cellulose-degrading bacterium isolated from soil. Int J Syst Evol Microbiol 2024; 74:6331.

Huson, D. H. & Scornavacca, C. Dendroscope 3: An interactive tool for rooted phylogenetic trees and networks. Syst. Biol. 61, 1061–1067 (2012).

Katoh, K. & Standley, D. M. MAFFT multiple sequence alignment software version 7: Improvements in performance and usability. Mol. Biol. Evol. 30, 772–780 (2013).

Lee B, Shin D, Kim J, Shin SK, Yi H, Baek MG. Massilia litorea sp. nov., Marinobacter salinisoli sp. nov. and Rhodobacter xanthinilyticus sp. nov., isolated from coastal environments. Int J Syst Evol Microbiol 2024; 74:6255.

Nylander, J. A. A. MrModeltest v2 (Uppsala University, 2004).

Oren A, Göker M. Validation list no. 218. List of new names and new combinations previously effectively, but not validly, published. Int J Syst Evol Microbiol 2024; 74:6398.

Ronquist, F. & Huelsenbeck, J. P. MrBayes 3: Bayesian phylogenetic inference under mixed models. Bioinformatics 19, 1572–1574 (2003).
